# The application of microfluidic technology in allergen detection: A review

**DOI:** 10.1097/MD.0000000000042645

**Published:** 2025-06-06

**Authors:** Dandan Luo, Peng Wang, Huifei Lu, Xianhai Zeng, Hailiang Zhao, Jiangqi Liu

**Affiliations:** a Department of Operating Room, The Affiliated Hospital of Zunyi Medical University, Zunyi, Guizhou, China; b Department of Graduate and Scientific Research, Zhuhai Campus of Zunyi Medical University, Zhuhai, China; c Department of Otolaryngology, Shenzhen Key Laboratory of Otolaryngology, Shenzhen Institute of Otolaryngology, Shenzhen Longgang Otolaryngology Hospital, Shenzhen, China.

**Keywords:** allergen, allergic diseases, microfluidic, microfluidic chips

## Abstract

The escalating prevalence of allergic diseases has intensified the need for rapid and precise allergen detection, prompting the exploration of microfluidic technology in this domain. This review delves into the utility of microfluidics in enhancing allergen detection, underscoring its high sensitivity, throughput, and miniaturization advantages. We survey the spectrum of microfluidic chip materials, from silicon wafers to paper-based substrates, and discuss various fabrication techniques. The integration of microfluidics with detection methods like enzyme-linked immunosorbent assay (ELISA), polymerase chain reaction, Western blotting, loop-mediated isothermal amplification (LAMP), and biosensors has transformed allergen diagnostics, offering more efficient, sensitive, and user-friendly platforms. These advancements facilitate point-of-care testing and hold significant potential for personalized medicine. Our review highlights the novel applications of microfluidics in allergen detection, contributing valuable insights for the development of future diagnostic tools and clinical research in allergy treatment. The innovative convergence of microfluidics with existing diagnostic methods presents a paradigm shift in the field of allergen detection, promising enhanced prevention and management of allergic diseases.

## 1. Introduction

The global surge in allergic diseases has become a public health epidemic, with escalating incidence rates that are closely linked to environmental changes and modern lifestyles. According to the World Health Organization, allergic diseases, including asthma, rhinitis, and eczema, affect an estimated 30% to 40% of the global population, imposing a substantial socioeconomic burden due to increased healthcare costs and reduced quality of life.^[1,2]^ The economic impact is further exacerbated by the significant rise in mortality rates associated with severe allergic reactions.^[3]^ Identifying and avoiding allergens is recognized as the most effective strategy for the prevention and management of these diseases.^[4]^ However, current diagnostic methods, which often rely on large-scale laboratory equipment and specialized facilities, are plagued by high costs, lengthy procedures, and a propensity for errors such as false positives and cross-contamination.^[5]^ These limitations underscore an urgent need for more efficient and accurate allergen detection methods. Therefore, quickly and accurately detecting allergens has gradually become an increasingly important issue for researchers.^[6]^ However, despite the wealth of literature on allergen detection, a gap remains in the comprehensive review of microfluidic technology’s role in this field. While several studies have explored individual aspects of microfluidics in diagnostics, a synthesized understanding of its applications, materials, and detection methods in allergen detection is lacking. This review aims to fill this void by providing a holistic overview of microfluidic technology’s application in allergen detection, assessing its potential to revolutionize the diagnosis and treatment of allergic diseases.

Microfluidics, also known as micrototal analysis systems, involves the complete exploration of the tiny dimensions and dimensions of nanoliter or picoliter fluid channels ranging from tens to hundreds of micrometers in size. This new interdisciplinary project involves physics, chemistry, microelectronics, biology, and biomedical engineering and is also known as a microfluidic chip due to its integration and miniaturization.^[7]^ Originally designed for chromatography, electrophoresis, and printing techniques, this method has been used in a range of fields, including engineering, chemistry, environment, existence sciences, organic research, and medical diagnostics.^[8,9]^ When microfluidic technological information is set up to gather a large variety of allergen molecules on a chip, quick serological detection is then carried out by sensitizing immunoglobulin E (IgE) with a small quantity of serum.^[10]^ Advances in microfluidic technology have permitted the design and development of miniaturized devices to control, coordinate, and modify microfluidics through 10 to 100-μm microchannels, which are now acting as microreactors to detect pathogenic microorganisms^[5]^ and creatinine,^[11]^ coronavirus disease 19 (COVID-19) testing,^[12]^ lab-on-a-chip,^[13]^ and organ-on-a-chip^[14]^ also rely on their use. An increasing number of detection applied sciences are built on a small chip based on a microfluidic device as a substitute for ordinary large-scale experimental tools and laboratories to obtain the speedy and point-of-care (POC) detection of allergens.^[15]^ This paper reviews the commonly used techniques of microfluidic chips, the materials used and the application of microfluidics in allergen detection. It provides valuable insights for our further research into more efficient and accurate allergen detection methods, as well as a comprehensive theoretical reference for clinical research into the treatment of allergic diseases.

## 2. Materials and fabrication technology of the microfluidic chip

The choice of the optimum material for microfluidic chip device fabrication is one of the fundamental steps in microfluidic applications. The choice must consider durability, ease of fabrication, transparency, biocompatibility, chemical compatibility with the implied reagents, ability to meet the temperature and pressure conditions needed for the reaction, and potential for surface functionalization.^[16,17]^ At present, commonly used substances for microfluidic chips include silicon wafers, glass, polydimethylsiloxane (PDMS), polymethyl methacrylate, polytetrafluoroethylene, and paper-based materials.^[18–25]^ Each material has its advantages and disadvantages, and the many manufacturing substances and mixtures of these substances vary greatly in terms of light transmittance, thermal conductivity, stability, hardness, manufacturing price, and application (Table 1).

**Table 1:** Comparison of several materials for the fabrication of microfluidic devices.

Author	Materials	Advantages	Disadvantages	Application
Yadavali et al^[18]^ 2018	Silicon–glass	Good light transmittance and stability	High cost	DNA amplification and biochemical reactions
Funano et al^[19]^ 2021	Glass–glass	High transparency, good stability, hardness, and biological inertness	The process is complex and the cost is high	Cell culture
Li et al^[20]^ 2023	Glass	High hardness, good light transmittance, relatively low cost	Poor thermal conductivity, no pressure resistance	Cell sorting techniques, immunoassay
Shakerit et al^[21]^ 2021	PDMS	High transparency, easy molding, biocompatibility, and air permeability	High production cost	Cell proliferation and differentiation
Abate et al^[22]^ 2020	Paper base-PMMA	Low cost and easy to operate	Rough manufacturing process	PCR
Feng et al^[23]^ 2020	PTFE-Silicone oil coating	It can be used with irritating organic solvents	Lack of transparency	Connectivity between microfluidic chips
Grover et al^[24]^ 2008	Teflon film	Transparent membrane, resistant to almost all chemical agents	–	Chemically inert monolithic membrane valves and pumps in microfluidic devices
Ozefe et al^[25]^ 2023	Paper base	Low cost and consumption	Easy to damage	In vivo culture

DNA = deoxyribonucleic acid, PCR = polymerase chain reaction, PDMS = polydimethylsiloxane, PMMA = polymethyl methacrylate, PTFE = polyethylene.

Different materials have different physical and chemical properties, therefore, the fabrication strategies and processes must follow the specific characteristics of the material.^[26]^ With the rapid improvement in the application of microfluidic chip technology, researchers have developed various microfluidic chip fabrication methods, such as photolithography,^[27]^ micromachining,^[28]^ molding/casting,^[29]^ laser ablation,^[30]^ 3D printing,^[31]^ and chemical etching.^[32]^ Each fabrication method has advantages and drawbacks, and researchers can use these methods alone or in combination according to product requirements and costs.

## 3. Application of microfluidic technology in allergen detection

**Figure 1.**
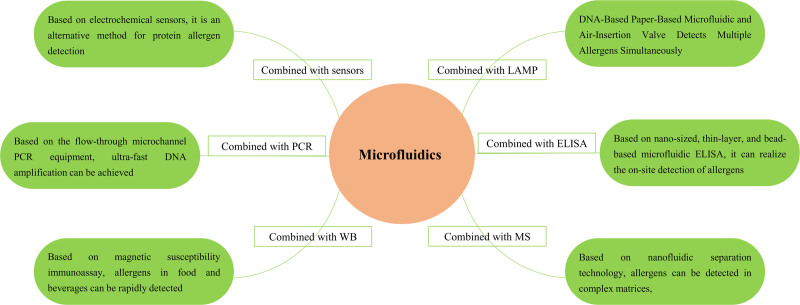
Microfluidic technologies combined with different allergen detection methods in POC technologies. POC = point-of-care.

Because of its ability to analyze various samples, including blood, saliva, or cell tissues, microfluidic technology has become firmly established in the fields of immunoassays, biochemical analysis, and molecular diagnosis. In a range of POC detection platforms, fabricating microfluidic chips with multiple microfluidic channels and integrating these chips with ultrasensitive detection techniques allows for multiplex evaluations with high throughput and high sensitivity.^[33]^ Microfluidic technology has a precise correlation with standard laboratory techniques in the differential prognosis of allergen sensitivity.^[34]^ Therefore, a combination of microfluidic technologies with different allergen detection methods can be used for POC technologies (Fig. 1). Below is an overview of the use of microfluidics in combination with a range of allergen detection strategies.

### 3.1. Application of microfluidic technology combined with ELISA in allergen detection

With the traits of excessive sensitivity, excessive specificity, convenient operability, repeatability, quick detection speed, and excessive throughput, enzyme-linked immunosorbent assay (ELISA) is broadly used in the quantitative evaluation of goal proteins and is an internationally identified standardized diagnostic approach,^[35]^ specifically appropriate for the detection of massive portions of samples. Therefore, ELISA is one of the most commonly used techniques for allergen detection. However, typical ELISA experiments require more than 1 washing step and a lengthy incubation time for every response step, making the detection laborious and time-consuming (usually half of a day in total). The detection website is concentrated in a giant laboratory, requires giant detection equipment, has a long operation time, passes illness for the duration of the detection process, and is associated with false-positive or false-negative results. It is difficult to gain fast and correct detection in this field.^[36]^

To resolve this problem, in recent years, researchers have built-in protein microarrays for allergen-specific antibody detection by incorporating microfluidic technological know-how into microfluidic chips, imaging chemiluminescence as an evaluation technology, mixing microfluidic technology with ELISA detection, and developing a collection of microfluidic chip fast detection systems. Heyries et al^[37]^ used a microfluidic device modified with apple, hazelnut, and pollen allergens to detect the serum of allergic sufferers and demonstrated the feasibility of a small chemiluminescence ELISA. Nakao et al^[38]^ proposed a thin-layer microfluidic ELISA as a new platform primarily based on the original nanomicrofluidic ELISA. Through verification, thin-layer ELISA may further improve the detection sensitivity with the aid of similarly enhancing the low-depth S/V ratio. Parent et al^[39]^ demonstrated the feasibility of capturing and cleaning steps in inner microfluidic chips by designing a regularly occurring microfluidic platform for the integration of complicated biotechnology by processing magnetic beads (MBs) grafted with trapping molecules. This study was performed by combining an entire ELISA protocol with calibration and washing steps to strengthen 2 microfluidic chip architectures. In addition, a complete ELISA protocol with built-in optical detection was developed, and the effects of protein quantification could be measured within 1 hour. The feasibility of this system was verified in food allergen assays with final protein quantification in a dynamic range of 10 to 30 ppm with a sensitivity of 2 ppm, and different allergens can also be quantified via the same microfluidic platform. Therefore, the combination of microfluidics with a typical ELISA makes allergen detection easier, more sensitive, and convenient and offers great application possibilities for POC detection of allergens. However, the total wash and sample volume of the system can be limiting.

### 3.2. Application of microfluidic technology combined with PCR in allergen detection

Polymerase chain reaction (PCR) is a molecular biology method used to enhance unique deoxyribonucleic acid (DNA) fragments and is extensively used in different fields, such as molecular biology, pathogen detection, clinical diagnostics, and meal testing.^[40]^ However, the challenges to the promotion of PCR for immediate detection are related to its speed, convenience, complexity, and even cost. The most essential characteristic of PCR is that it can greatly increase the quantity of DNA fragments. With PCR, any nucleic acid sequence in a complicated pattern can be amplified in a cycle.^[41]^ However, due to the inactivation of DNA polymerase at excessive temperatures, new DNA polymer has to be brought to every cycle, which is cumbersome, pricey, and has a lengthy response time. Therefore, these dangers of DNA polymerase limit the application and improvement of PCR technology. A portable all-in-one microfluidic system based entirely on a built-in CF-PCR and electrophoresis biochip robotically injects assay samples into the chip, which is a technique that can change luxurious exterior precision syringe pumps. With this technique, dense helix amplification is as quick as 2'31'' and PCR product detection is carried out at 3'43'',^[42]^ it no longer solely realizes speedy DNA amplification and on-site PCR product detection, however, additionally computerized pattern injection. This built-in machine can be utilized for instant allergen detection for allergic diseases. A closed loop is a wonderful microfluidic reactor (μPCR) that makes use of round microchannels, thereby permitting DNA samples to continuously pass through unique temperature zones to entire the PCR cycle. closed loop μPCR has the fundamental advantages of normal continuous flow μPCR while eliminating most of the negative aspects related to lengthy serpentine microchannels. A 3-dimensional warmth switch mannequin was used to calculate the temperature distribution inside the microreactor, and the dwelling time was extracted in accordance with this distribution. The calculations confirmed that for the best-performing microreactor design, 30 cycles of PCR were performed in less than 3 minutes. Skaltsounis fabricated PCR chips primarily based on the calculations, measured the temperatures on their surfaces by using thermography, and then compared the measurements with the calculations to validate the accuracy of the microreactor model.^[43]^

The PCR chips enable the integration of microheaters while being low-cost, reliable in terms of heating temperature, and suitable for mass production. However, business benchtop PCR lacks a quick and secure heating and cooling platform, and the detection time requires 30 to 40 minutes. A current study developed an ultrafast PCR platform with the use of a microchannel chip.^[44]^ The very quick warmness of the reagents on this platform and the easy design and fabrication of the chip make it appropriate for the improvement of industrial ultrafast PCR chips. Currently, this ultrafast PCR platform is in the development stage and has not yet been validated and applied in allergen detection. Therefore, this ultrafast PCR platform must be further validated and improved.

### 3.3. Application of microfluidic technology combined with WB for allergen detection

As an emerging assay, Western blotting (WB) can be used for semiquantitative and qualitative evaluations of proteins in more than a few allergens to be tested. Therefore, WB has been extensively used in proteomics research,^[45]^ but this method requires long evaluation time and more than 1 guide step. To increase automation and limit evaluation time, pattern volume, and reagent consumption for protein immunoblotting, microfluidic structures are being explored in a mixture with WB to improve the shortcomings of regular immunoassays. Microfluidic WB^[46]^ (μWestern) is carried out in an enclosed straight glass microfluidic channel with electrophoretic manipulation of all detection phases, offering mere electronic operation and fluidic manipulation bringing the want for pumps or valves, and is appropriate for examining a wide molecular weight vary 6.5 to 116 kDa. Microimmunodetection with direct deposition of immunodetection reagents^[47]^ allows the whole protein immunodetection method to be performed in <1 hour, with equal sensitivity as an incubation step requiring 20 hours. The use of a low microliter/min drift rate allows the antibody reagent to be utilized immediately in the membrane where the goal protein is captured, resulting in up to a 30-fold discount in antibody consumption, which improves the velocity of detection and miniaturization of the WB.

In addition, PDMS is commonly used in the preparation of microfluidics and polyacrylamide gel electrophoresis (PAGE), in which a network of PDMS microchannels is reversibly bound to the slide to allow direct access to the isolated proteins, accompanied by diffusion-controlled in situ immunostaining and Sypro Red staining of total proteins.^[48]^ This PAGE-in-PDMS fabrication technique extends the application of microfluidic PAGE without the need for glass micromachining infrastructure. However, PAGE has limited resolution for native proteins and relatively slow separation times, which can be overcome by microfluidic thermal transient isotachophoresis. Microfluidic thermal gel isotachophoresis with transient speed^[49]^ uses a thermogel as the separation matrix, and because thermogels have the special capacity to change viscosity in response to modifications in temperature, proteins can be delivered to the thermogel and loaded into a microfluidic gadget to gain rapid, high-performance separation of native proteins. Compared with native PAGE, this approach offers a large mass range and 2-fold greater resolution while requiring 15,000-fold less protein loading and a 5-fold discount in analysis time.

Hughes et al^[50]^ investigated a fully microfluidic WB, which modernizes and automates traditional WB, taking advantage of precision and management afforded by way of integrating microfluidics to completely capture and evaluate the sample. All steps, from sample separation to detection, are completed in a short period, lowering the equipment run time and footprint, reducing reagent costs, and permitting a quantitative readout of information generated by multiple analytical assays in a single pattern and 48 blot chips. A disposable electrochemical microfluidic system primarily based on the susceptibility immunoassay approach developed by Baldo et al^[51]^ can be used for the rapid and easy detection of different proteins that act as allergens in meals and beverages, with effects consistent with those of ELISA. The mixture of microfluidics permits fast and touchy WB detection and the ability to examine records from multiple analyte assays at identical times, both of which are vital for the future cognizance of immediate onsite detection of allergens.

### 3.4. Application of microfluidic technology combined with LAMP in allergen detection

A novel technique for amplifying nucleic acids is called loop-mediated isothermal amplification (LAMP). A molecular biology technique called nucleic acid amplification is used to quickly amplify particular DNA fragments in vitro. LAMP technology is easier, quicker, and more precise than traditional PCR.^[52]^ However, LAMP technology is only capable of detecting allergens to a limited extent, as the extraction of nucleic acids from a single plant seed is comparatively complex. In light of this, Sun et al^[53]^ extracted and purified nucleic acids from plant seed allergens and commercial items with untreated glass fiber paper (paper-based microfluidics). LAMP amplification can be immediately used for this paper-based DNA microfluidics technology. Paper-based microfluidics with LAMP offers good specificity, repeatability, and sensitivity. They can detect allergens quickly and sensitively, according to amplification data. Furthermore, the procedure facilitates an integrated approach to nucleic acid detection on paper, which includes extraction, setup, and preparation steps for LAMP experiments. Consequently, allergens in commercial items can be extracted and detected via the paper-based chip nucleic acid extraction approach in conjunction with LAMP detection.

Natsuhara et al^[54]^ developed a microfluidic diagnostic device with an air inlet valve for the simultaneous detection of different food allergens. The results confirmed that a variety of flour allergens in the mixture of wheat DNA and buckwheat DNA could be detected simultaneously with the LAMP assay without any cross-contamination. Yuan et al^[55]^ developed a colorimetric LAMP microfluidic chip by combining LAMP with a microfluidic chip, which can detect 3 allergens, peanut, sesame, and soybean allergens, simultaneously in 1 chip via the naked-eye colorimetric method. Research results show that the combination of paper-based microfluidics and LAMP makes the detection of allergens faster, more sensitive, and more convenient, and there is no cross-contamination in the detection process, it can detect a large number of allergens simultaneously, which represents a new method for the immediate diagnosis of allergic diseases. Although many studies on microfluidics based on LAMP have been conducted, the current studies target only a few common allergens, and most of them are in the verification phase. To achieve large-scale screening of allergens, many experiments must be conducted to verify them in future research.

### 3.5. Application of microfluidic technology combined with biosensors in allergen detection

Biosensors are widely used in biochemistry, electrochemistry, agriculture, and biomedicine.^[56]^ Traditional biosensors usually use antibodies as recognition molecules, but the instability and high cost of antibodies limit their application. This problem has been solved by the development of microfluidics, which harness hydrodynamics to drive miniaturized biosensors with the advantages of mobility, operational transparency, controllability, and stability. The convergence of microfluidics and biosensor technology has enabled the precise control of volumes as small as 1 nanoliter and the integration of various types of bioassays into a miniaturized platform.^[57]^ This integration offers several advantages, such as low reagent consumption, automatic sample preparation, shorter processing time, low analysis costs, minimal handling of hazardous substances, high test accuracy, portability, and usability. The microfluidic cell chip, which was originally used to monitor allergic reactions, consists of a cell culture chamber and a microfluidic channel into which the fluorescent dye quinacrine is introduced into the cell chamber, and the fluorescent signal of quinacrine is detected by a microfluidic system.^[58]^

In a later study, Jiang developed a novel intercellular electrochemical microfluidic chip for the qualitative and quantitative analysis of food allergens, which enables simultaneous microfluidic cell culture, food allergen-induced changes in cell morphology, and cell metabolism.^[59]^ The chip enables accurate monitoring of cellular allergic responses in real time and provides a general example of the rapid development of cost-effective biosensor technologies for food allergen detection and research. In recent years, the demand for the integration of biosensors into micro- and nanofluidic-based lab-on-a-chip devices for POC diagnostics has increased.^[60]^ The compatibility of plastic microfluidic French fries based on functionalized MBs can be integrated into POC diagnostics and has been shown to be comparable to conventional ELISAs performed by clinicians in the laboratory and compatible with other antibody assays obtained directly from patient samples.^[61]^ With the development of nanotechnology, the application of nanotechnology and microfluidics in electrochemical biosensors has also greatly improved their detection performance^[62]^ and has been successfully applied in the field of allergen detection. Jiang et al^[63]^ developed an origami microfluidic electrochemical nanosensor for the rapid detection of the peanut allergen Ara h1. The sensor could perform the test in 20 minutes with a linear range of 50 to 1000 ng/mL and a detection limit of 21.6 ng/mL. This combination of microfluidic electrochemical nanosensors shortened complex laboratory tests and food allergen analysis and significantly reduced the testing time.

A microfluidic paper biosensor developed by Ramalingam was specific for the presence of gentamicin in milk samples.^[64]^ This method is specific for the presence of gentamicin in milk samples and can be used to calculate the amount of gentamicin in a sample within 2 minutes. Bifunctional core shells of blue nanoparticles and nanoenzymes were combined with a microfluidic device, a smartphone, and a portable Raman spectrometer for portable detection of food allergy proteins.^[65]^ This method not only replaces natural peroxidase for immunochromatographic detection but also serves as a unique Raman marker for surface-enhanced Raman scattering analysis, which has great potential for practical applications in allergen detection. Moreover, Michailia et al^[66]^ developed a system that combines a silicone-based microsensor chip and an advanced microfluidic module, which has been shown to be capable of detecting 4 unlabeled allergens simultaneously, with results that are consistent with those of ELISA. The present study is based on the analytical properties of this sensor, which, together with the short analysis time, small chip size, more than 10-fold reusability of the chip, and relatively low operating costs, make it an ideal tool for on-site analysis and identification of a wide range of allergens.

### 3.6. Application of microfluidic technology combined with MS in allergen detection

Mass spectrometry (MS) is characterized by high sensitivity and structural analysis and is particularly suitable for the detection of allergens with unknown structures. Although ELISA is widely used for the detection of serum allergens, it is prone to false-positive or false-negative results. Therefore, more accurate validation methods are needed. MS can provide a more reliable method for confirming allergen uptake than can ELISA and offers a rigorous alternative to allergen assays.^[67]^ The MS technique, combined with microfluidic separation as an analytical tool, enables the detection and quantification of food allergens via a simple extraction method with results consistent with those of ELISA. Rebekah et al^[68]^ reported that microfluidic separation technology improves sensitivity and ionization efficiency at low concentrations and that targeted MS enables the detection of allergens causing allergic diseases in food matrices in the range of 10 to 50 mg/kg arachidonic acid. Immunoaffinity capillary electrophoresis (IACE) in combination with matrix-assisted laser desorption ionization MS (MAL-DI/MS) can be used to differentiate allergenic components in milk. Only 2 μL of serum is needed. Total IgE quantification and component-resolved diagnostic for food allergies can also be performed directly via food extracts.^[69]^ This method offers the possibility of direct identification of the molecular mass and structure of allergens as well as the discovery of unknown allergens and is useful for accurate personalized allergy diagnosis, mapping of allergen epitopes and cross-reactivity studies.

Zhao et al^[70,71]^ proposed a protocol for the detection of allergens in seafood by combining microfluidic French fries with matrix-assisted laser desorption ionization time-of-flight MS (MALDI-TOF MS). The protocol investigated the nonspecific adsorption of commercial MBs to biomolecules and the reproducibility and sensitivity of the protocol, and the results were consistent with those of the ELISA. Allergens in food matrices are well detected by immunomagnetic IgE separation from blood samples via commercial MBs with antihuman IgE antibody functionality, followed by the capture of allergens in protein extracts from seafood in a single direct microfluidic channel and the digestion and characterization of the captured allergens via MALDI-TOF MS and high-performance liquid chromatography–tandem MS. With a false-positive rate of 0%, this protocol can be used not only to identify unknown seafood allergens and patient serum allergenicity, but also to predict allergic cross-reactivity between fish, to counsel individuals with a history of seafood allergy, and to ensure the safety of patients with food allergies.

In addition, microfluidic microarrays in combination with mass spectrometric detection technology can not only separate complex mixtures, but also identify individual components.^[72]^ IgE in the blood is a class of antibody that is associated mainly with allergic reactions. The separation of allergens in exudate samples via IgE antibody-modified MBs allows potential allergens in the samples to be captured by the IgE-biofunctional MBs in the microfluidic channel and the MBs bound to the proteins to be separated under the influence of a magnetic field. The allergens are digested and then analyzed via matrix-assisted laser desorption/ionization time-of-flight MS. A microfluidic magnetic-based allergen extraction scheme has been proposed for the rapid detection of allergens in seafood.^[73,74]^ This method can detect 100 allergens in seafood and offers a new rapid detection method for multiple allergens. However, as food processing and storage can lead to the denaturation of plant proteins in food, further research is needed to better standardize proteomics workflows for food allergen analysis.

## 4. Conclusion

In conclusion, the integration of microfluidic technology in allergen detection has marked a significant advancement in the field of allergy diagnostics. This review highlights how microfluidic systems enhance sensitivity, specificity, and rapidity in allergen detection, offering a transformative approach over traditional methods. The innovative applications of microfluidics, combined with techniques like ELISA, PCR, LAMP, and biosensors, provide a powerful platform for POC diagnostics. These advancements address critical gaps in rapid allergen identification and have the potential to revolutionize how allergic diseases are managed and treated. Despite the progress, the field is still burgeoning, and further research is essential to standardize microfluidic detection methods and expand their scope to a broader range of allergens. Looking ahead, the synergy of microfluidics with emerging technologies such as artificial intelligence and nanotechnology is poised to redefine large-scale allergen detection and diagnosis, heralding a new era in allergy management.

## Author contributions

**Conceptualization:** Jiangqi Liu.

**Funding acquisition:** Xianhai Zeng.

**Supervision:** Xianhai Zeng, Hailiang Zhao.

**Writing – original draft:** Dandan Luo, Peng Wang.

**Writing – review & editing:** Huifei Lu.
